# Cisplatin Nephrotoxicity and Longitudinal Growth in Children With Solid Tumors: A Retrospective Cohort Study

**DOI:** 10.1097/MD.0000000000001413

**Published:** 2015-08-28

**Authors:** Clímaco Andres Jiménez-Triana, Osvaldo D. Castelán-Martínez, Rodolfo Rivas-Ruiz, Ricardo Jiménez-Méndez, Aurora Medina, Patricia Clark, Rod Rassekh, Gilberto Castañeda-Hernández, Bruce Carleton, Mara Medeiros, the Canadian Pharmacogenomics Network for Drug Safety Consortium

**Affiliations:** From the Departamento de Nefrología, Hospital Infantil de México Federico Gómez (CAJ-T); Unidad de Investigación en Epidemiología Clínica, Hospital Infantil de México Federico Gómez (ODC-M, PC); Coordinación de Investigación en Salud, Instituto Mexicano del Seguro Social (RR-R); Facultad de Medicina de la Universidad Nacional Autónoma de México, México D.F., México (RR-R, PC); Pharmaceutical Outcomes Programme, BC Children's Hospital (RJ-M, BC); Division of Translational Therapeutics, Department of Paediatrics, Faculty of Medicine, University of British Columbia (BC, RJ-M); Child&Family Research Institute, Vancouver, Canada (RJ-M, BC); Departamento de Oncología, Hospital Infantil de México Federico Gómez, México D.F., México (AM); Department of Oncology, BC Children's Hospital Vancouver, Canada (RR); Departamento de Farmacología, CINVESTAV IPN (GC-H); Laboratorio de Investigación en Nefrología y Metabolismo Mineral, Hospital Infantil de México Federico Gómez (MM); and Departamento de Farmacología, Facultad de Medicina UNAM, México D.F., México (MM).

## Abstract

Cisplatin, a major antineoplastic drug used in the treatment of solid tumors, is a known nephrotoxin. This retrospective cohort study evaluated the prevalence and severity of cisplatin nephrotoxicity in 54 children and its impact on height and weight.

We recorded the weight, height, serum creatinine, and electrolytes in each cisplatin cycle and after 12 months of treatment. Nephrotoxicity was graded as follows: normal renal function (Grade 0); asymptomatic electrolyte disorders, including an increase in serum creatinine, up to 1.5 times baseline value (Grade 1); need for electrolyte supplementation <3 months and/or increase in serum creatinine 1.5 to 1.9 times from baseline (Grade 2); increase in serum creatinine 2 to 2.9 times from baseline or need for electrolyte supplementation for more than 3 months after treatment completion (Grade 3); and increase in serum creatinine ≥3 times from baseline or renal replacement therapy (Grade 4).

Nephrotoxicity was observed in 41 subjects (75.9%). Grade 1 nephrotoxicity was observed in 18 patients (33.3%), Grade 2 in 5 patients (9.2%), and Grade 3 in 18 patients (33.3%). None had Grade 4 nephrotoxicity. Nephrotoxicity patients were younger and received higher cisplatin dose, they also had impairment in longitudinal growth manifested as statistically significant worsening on the height Z Score at 12 months after treatment. We used a multiple logistic regression model using the delta of height Z Score (baseline-12 months) as dependent variable in order to adjust for the main confounder variables such as: germ cell tumor, cisplatin total dose, serum magnesium levels at 12 months, gender, and nephrotoxicity grade. Patients with nephrotoxicity Grade 1 where at higher risk of not growing (OR 5.1, 95% CI 1.07–24.3, *P* = 0.04). The cisplatin total dose had a significant negative relationship with magnesium levels at 12 months (Spearman *r* = −0.527, *P* = <0.001).

## INTRODUCTION

The success in cancer therapy has increased the remission rate and survival time of patients, it is now common that cancer survivors face the chronic effects of treatment such as ototoxicity, neurotoxicity, nephrotoxicity, and skeletal dysfunction.^1–6^

Cisplatin is a commonly used antineoplastic drug in the treatment of solid tumors. It binds to deoxyribonucleic acid (DNA), leading to intra- and interstrand cross-links that result in defective DNA templates and interfere with DNA synthesis and replication.^7,8^ It also affects mitochondria by inhibiting ATPase activity and causing apoptosis, oxidative stress, inflammation, and necrosis. It can be ototoxic, myelotoxic, gastrotoxic, neurotoxic, and nephrotoxic.^9,10^

In the kidney, the main damage occurs in the renal tubules.^11^ The most common adverse effects of tubular damage include hypomagnesemia, hypophosphatemia, hyponatremia, hypocalcemia, normoglycaemic-glucosuria, proteinuria, metabolic acidosis including Fanconi syndrome, and nephrogenic diabetes insipidus. Cisplatin can also produce glomerular damage (thrombotic microangiopathy) and acute kidney injury (AKI) as a result of acute tubular injury/necrosis, which can further progress to chronic kidney disease.^3,12^

Cytotoxic chemotherapy treatment has been linked to delayed longitudinal growth in children with cancer.^13^ Bone is the main repository of minerals in the body including calcium, phosphorus, magnesium, and trace elements. Bone formation is directly linked to longitudinal growth and in animal models cisplatin has shown to inhibit new bone formation.^14^ Data have also been published indicating that after standard cisplatin chemotherapy is completed, platinum exposure continues for at least 20 years. Whether chronically elevated platinum concentrations influence the development of these or other late toxicities including secondary malignancies is unknown.^15^

The aim of our study was to evaluate the prevalence and severity of cisplatin nephrotoxicity in children and to determine the impact of nephrotoxicity on growth.

## PATIENTS AND METHODS

### Study Participants and Design

Retrospective study of children treated with cisplatin for solid tumors that were part of a cohort studied for adverse reactions to chemotherapy, approved by the Hospital IRB and Ethics Committee at the Hospital Infantil de México Federico Gómez. This large public hospital receives patients referred from across the country. All patients or parents provided written informed consent.

Patients treated for childhood cancer (≤18 years of age at the start of therapy) received cisplatin therapy between June 2002 and May 2013.

The information about weight, height, serum creatinine, and electrolytes (magnesium, phosphorus, and potassium) before each cisplatin cycle and after 12 months of treatment was obtained from the medical record. We excluded patients with incomplete information.

The standard deviation score (Z Score) for height and weight was calculated with the app STAT GrowthCharts version 3.2 that uses the National Center for Health Statistics 2000 Center for Disease Control Growth Data.^16^ The Z Score was obtained by subtracting the actual patient weight or height from the mean weight or height of the population of that chronological age and gender divided by the appropriate standard deviation. A Z Score of 0 means that the patient has the average height or weight for age and gender. Values below −2.0 or +2.0 are outside normal growth charts.

Glomerular filtration rate (GFR) was estimated using Schwartz formula as serum creatinine was measured by Jaffe method.^17^ Hypomagnesemia was defined as Mg values <1.5 mg/dL. It was considered severe when Mg is ≤1.0 mg/dL.^18^ Hypophosphatemia was defined when phosphate values below age recommended values by the National Kidney Foundation clinical practice guidelines (KDIGO):^19^ 6 to 12 months 5.0 to 7.8 mg/dL; 1 to 5 years 4.5 to 6.5 mg/dL; 6 to 12 years 3.6 to 5.8 mg/dL; and 13 to 20 years 2.3 to 4.5 mg/dL. Hypokalemia was defined when potassium values below age recommended values:^20^ 1 month to 2 years 3.7 to 5.9 mEq/L; >2 years to 18 years 3.5 to 5.0 mEq/L.

### Nephrotoxicity Definition

There are no standards to identify drug-induced kidney disease. We decided to grade nephrotoxicity considering a score that combines AKI definition^21^ and tubulopathy, manifested as electrolyte disturbances as follows: normal renal function (Grade 0); asymptomatic electrolyte disorders (hypomagnesemia, hypokalemia or hypophosphatemia), including an increase in serum creatinine, up to 1.5 times baseline value (Grade 1); need for electrolyte supplementation (magnesium, potassium, or phosphate) <3 months and/or increase in serum creatinine 1.5 to 1.9 times from baseline (Grade 2); increase in serum creatinine 2 to 2.9 times from baseline or need for electrolyte supplementation (magnesium, potassium, or phosphate) for more than 3 months after treatment completion (Grade 3); and increase in serum creatinine ≥3 times from baseline or renal replacement therapy (Grade 4).

The decision to initiate electrolyte supplementation was made by the treating oncologist.

A clinical pharmacologist, a pediatric nephrologist, a pediatric oncologist, and an adverse drug reaction surveillance clinician performed the clinical characterization.

### Statistical Analysis

Descriptive statistics is reported as means and standard deviations or median (25th, 75th percentile) for continuous measures and percentages for binary/categorical measures. Differences between nephrotoxicity and nonnephrotoxicity patients were performed using Student's *t*-test, Mann–Whitney, or Fisher exact test depending upon the variable. Cisplatin total dose and nephrotoxicity grading was compared by Kruskal–Wallis test. Height and weight Z Score at baseline versus Z Score at 12 months of treatment was compared using paired *t*-test.

Repeated measures analysis of variance with Bonferroni post-test or Friedman test, depending upon the distribution of the variable, was used to analyze electrolyte, creatinine, and estimated GFR (eGFR) changes overtime.

Correlations between cisplatin dose and electrolyte levels at 12 months were determined by Pearson correlation coefficient. For all tests a *P* value <0.05 (2-sided) was considered statistically significant. All statistical analyses were performed using statistical analysis software (GraphPad Prism for MacOs X version 5.0).

For multivariate analysis we used a multiple logistic regression model using the delta of height Z score (baseline–12 months) as dependent variable in order to adjust for the main confounder variables such as germ cell tumor, cisplatin total dose, serum magnesium at 12 months, gender, and nephrotoxicity grade. We used the SPSS version 20 for Mac (IBM Corporation, Armonk, NY).

## RESULTS

### Patient Characteristics

**FIGURE 1:**
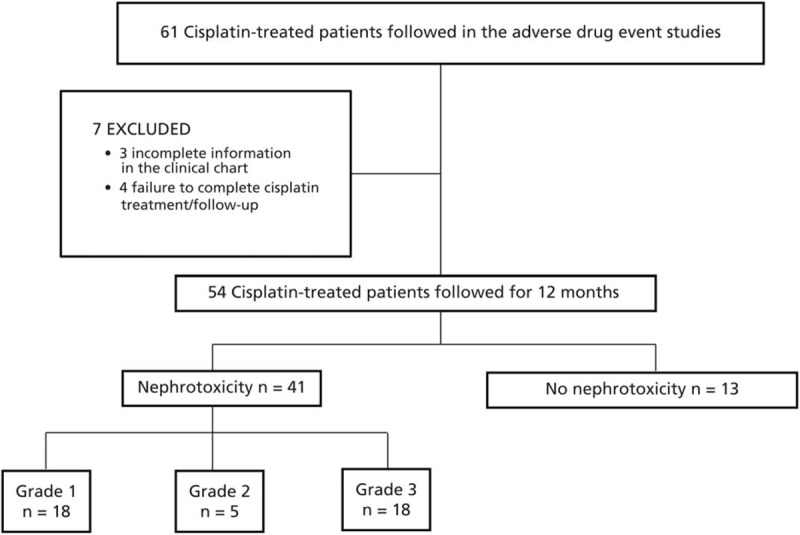
Flow diagram of the patients enrolled in the study.

We collected data on 61 cisplatin-treated patients that participated in the adverse drug event studies. Seven patients were excluded, 3 of them because of incomplete medical information in the clinical chart, and 4 because they did not complete cisplatin treatment. We have complete information for 54 patients (Figure 1). No patient received growth hormone.

### Nephrotoxicity

**FIGURE 2:**
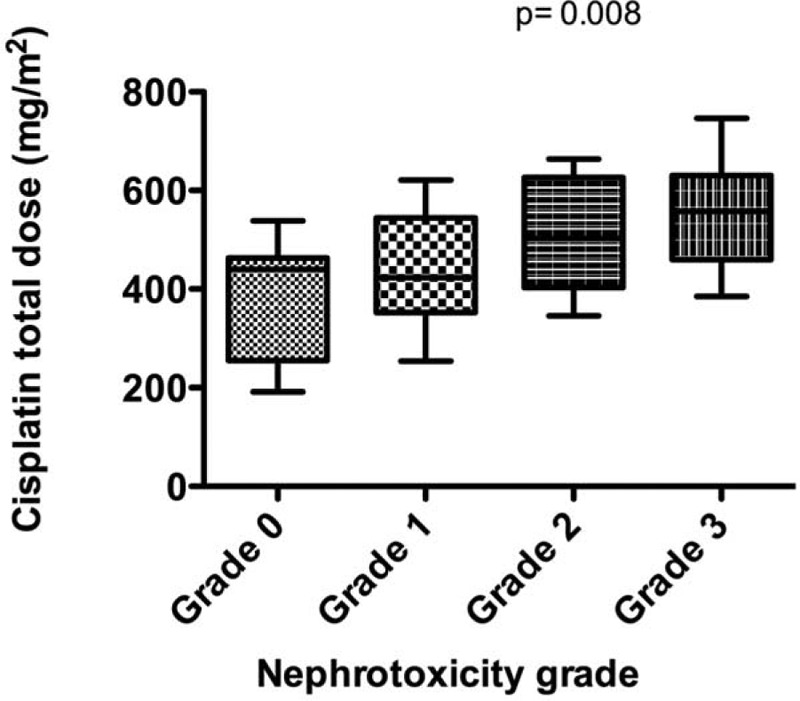
Cisplatin total dose and nephrotoxicity grade. Box and whisker plots show the 10th, 25th, 50th (median), 75th, and 90th percentile. The severity of nephrotoxicity increased with the cumulative dose (Kruskal–Wallis *P* = 0.008).

Patient demographics are outlined in Table 1. Nephrotoxicity was observed in 41 patients (76%). No nephrotoxicity (Grade 0) was observed in 13 patients (24%). Grade 1 nephrotoxicity was observed in 18 patients (33.3%), Grade 2 in 5 patients (9.2%), and Grade 3 in 18 patients (33.3%). None had Grade 4 nephrotoxicity. Only 5 patients showed an increase in serum creatinine, 4 of them 1.5 to 1.9 from baseline and only one 2.5 times from baseline, all of them required electrolyte supplements, one patient recovered to baseline serum creatinine at 12 months of follow-up. The severity of nephrotoxicity was related to the cisplatin total dose (Figure 2), Kruskal–Wallis *P* = 0.008.

**TABLE 1:**
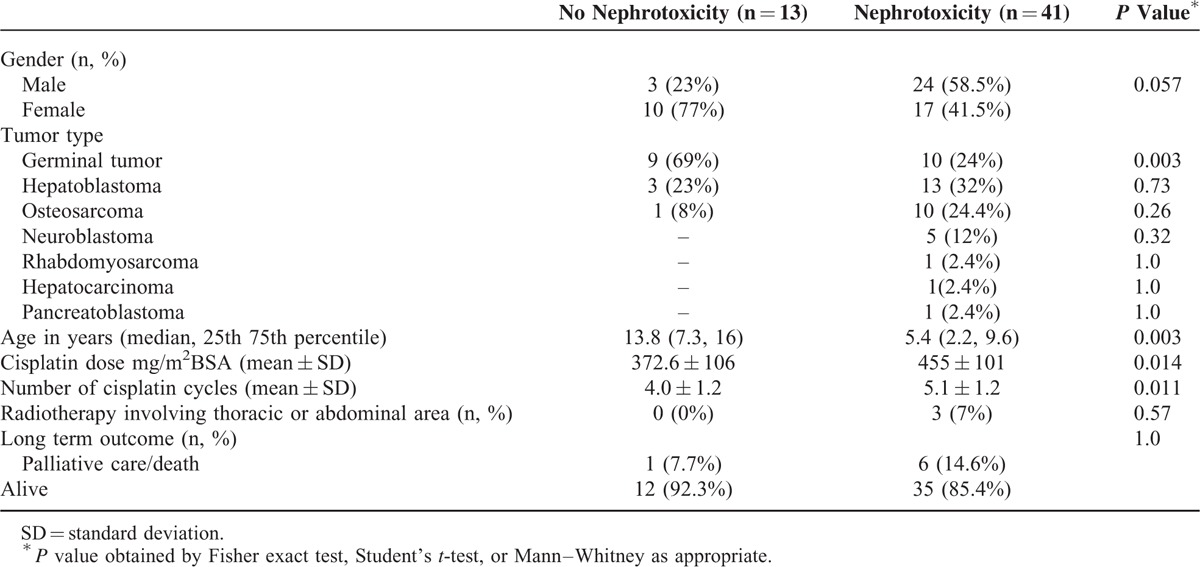
Patient Characteristics

Hypophosphatemia was found in 35 patients (65%), hypomagnesemia in 22 (40.7%), and hypokalemia in 20 patients (37%). Combined hypomagnesemia and hypophosphatemia was observed in 19 patients (35%) and 8 had hypomagnesemia, hypophosphatemia, and hypokalemia (14.8%).

Patients with nephrotoxicity were younger than patients with nonnephrotoxicity, median age 5.4 versus 13.8 years, respectively (*P* = 0.003), they received higher number of cisplatin cycles and therefore higher cisplatin total dose (mean accumulated dose in nephrotoxicity 455 versus 372 mg/m^2^ in nonnephrotoxicity, *P* = 0.01). Patients with nephrotoxicity had more diverse tumors than nonnephrotoxicity patients in whom germinal tumors were more frequent (Table 1).

### Concomitant Medications

There was no difference in the concomitant medications administered to nephrotoxicity and nonnephrotoxicity patients (Table 2). Three patients from nonnephrotoxicity and 7 patients from nephrotoxicity group received amikacin/cefipime twice during the 1st year of chemotherapy.

**TABLE 2:**
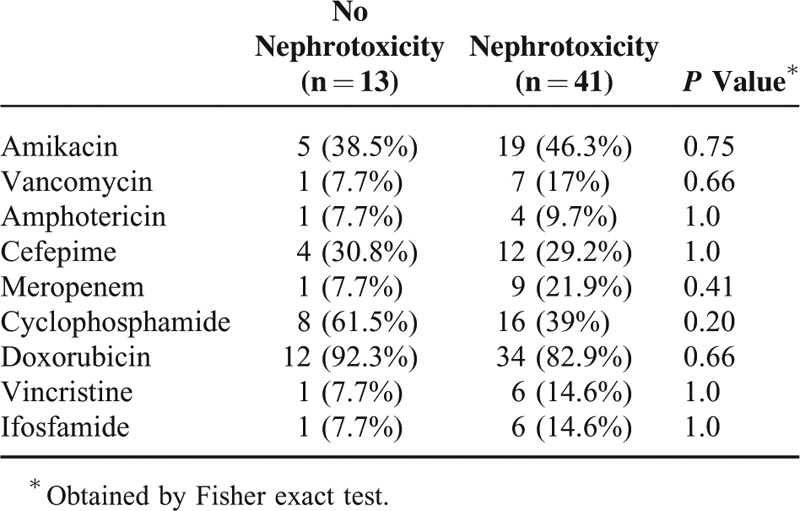
Cisplatin Nephrotoxicity and Concomitant Medications (n, %)

### Serum Electrolytes and Glomerular Filtration Rate Follow-up

Values of serum electrolytes, creatinine, and eGFR by Schwartz formula at baseline, 2nd, 3rd, 4th cisplatin cycle, and 12 months of follow-up are shown in Table 3. Phosphorus values showed a “U” shape overtime, starting with 4.43 ± 0.77 mEq/L, reaching the lowest value at the 3rd cisplatin cycle with 4.0 ± 0.90 mEq/L and the highest value at 12 months (4.74 ± 1.13 mEq/L). Phosphorus values were statistically significant different at the 2nd, 3rd, and 4th cisplatin cycles than at 12 months.

**TABLE 3:**
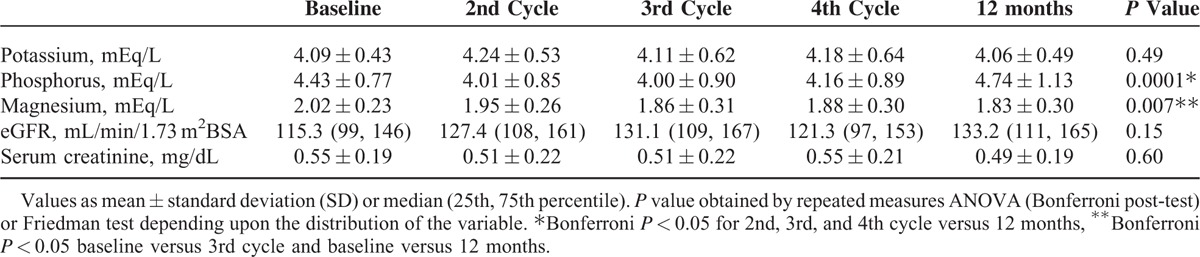
Cisplatin Cycle, Renal Function, and Electrolyte Levels

Magnesium level decreased each visit in a progressive way, being significantly lower at the 3rd cycle and at 12 months compared to baseline. Patients started cisplatin treatment with a magnesium level of 2.02 ± 0.23 and 1.83 ± 0.30 mg/dL at 12 months.

There was no statistically significant difference in serum potassium, creatinine, and eGFR. It is worth noting eGFR was not normally distributed and 12 patients had hyperfiltration (eGFR >150 mL/min/1.73 m^2^BSA).

### Weight and Height Z Score

**FIGURE 3:**
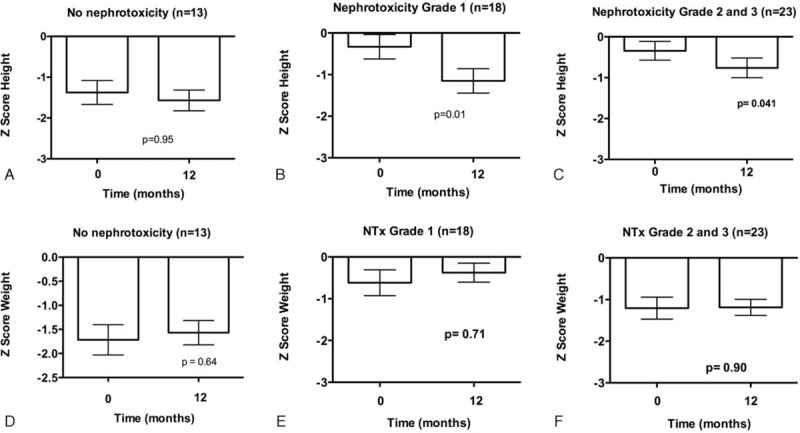
Nephrotoxicity grading and mean ± SE of height Z Score (A, B, C) and weight (D, E, F) at baseline and after 12 months of cisplatin treatment. *P* value obtained by paired *t*-test.

Patients without nephrotoxicity maintained their height Z Score after 12 months of treatment (baseline −1.37 ± 1.0, 12 months −1.56 ± 0.8, paired *t*-test *P* = 0.95, Figure 3A), whereas those with nephrotoxicity had worse height Z Scores after 12 months. Z Scores were more pronounced in those with nephrotoxicity Grade 1 (baseline −0.33 ± 1.2, 12 months −1.15 ± 1.1, *P* = 0.01, Figure 3B) than Grades 2 and 3 (baseline −0.34 ± 1.0 vs 12 months −0.8 ± 1.1, *P* = 0.04, Figure 3C). There was no difference in weight Z Score (basal vs 12 months) in all groups (Figure 3D–F).

The results of the multivariate logistic analysis of risk factors for the lack of longitudinal growth are shown in Table 4. Patients with nephrotoxicity Grade 1 where at higher risk of not growing (OR 5.1, 95% CI 1.07–24.3, *P* = .04).

**TABLE 4:**
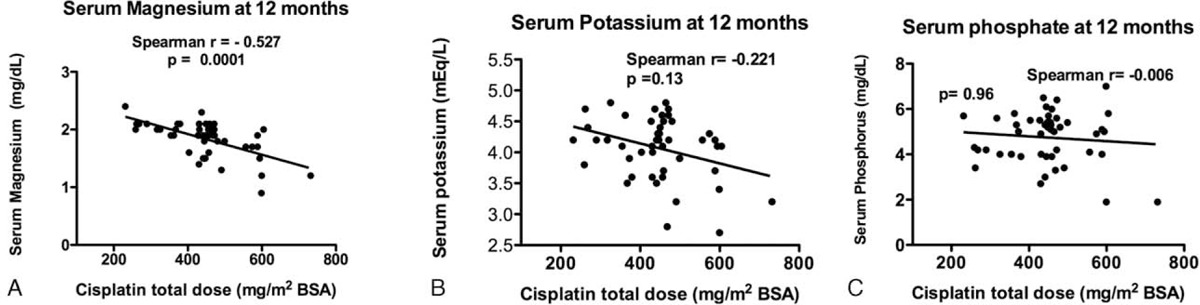
Multivariate Logistic Analysis of Risk Factors for the Lack of Longitudinal Growth at 12 months After Cisplatin Therapy

### Cisplatin Total Dose and Correlation With Electrolytes at 12 months

**FIGURE 4:**
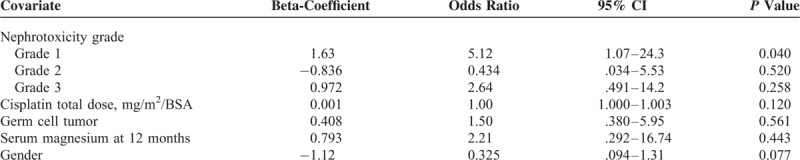
Correlation of cisplatin total dose and serum electrolyte levels at 12 months. (A) Magnesium, (B) potassium, and (C) phosphate.

The cisplatin total dose had a significant negative relationship with magnesium, potassium, and phosphate levels at 12 months, being statistically significant only for magnesium (Figure 4A, Spearman correlation −0.527, *P* = <0.001).

### Patient Follow-Up

After the 12 months, 1 patient in the nonnephrotoxicity (Grade 0) group was in palliative care, 4 patients in the group with nephrotoxicity died, and 2 went to palliative care.

## DISCUSSION

Our study confirms a high frequency (76%) of nephrotoxicity in cisplatin-treated children. In order to evaluate the cisplatin nephrotoxicity we propose a score that combines the AKI criteria considering the increase in baseline creatinine^21^ with an evaluation of the tubulopathy manifested as electrolyte disturbances, as this latter is the main and most frequent manifestation of cisplatin nephrotoxicity, but we included not only magnesium as Skinner et al have suggested^11,22^ but also phosphate and potassium.

Grade 1 (asymptomatic, nontreated electrolyte disturbances) and Grade 3 nephrotoxicity (increase in serum creatinine at 12 months 2–2.9 times from baseline or need for electrolyte supplementation for more than 3 months after treatment completion) had the same frequency, 33.3% in our study.

Nephrotoxicity patients received higher cisplatin dose as has been reported in other studies.^11,23^

An acute reduction in GFR with the respective increase in serum creatinine has been reported in 20% to 80% of cisplatin-treated children, with a GFR at 10 years after treatment <60 mL/min/1.73 m^2^ in 11%.^22^ In our study we found that 9.2% (5 patients) had an acute reduction in eGFR. Probably the proportion would be higher if we were able to use the new Schwartz bedside formula, a more precise equation to estimate GFR, unfortunately in our center serum creatinine is still measured by Jaffe method.

Hypomagnesemia has been pointed out as the most common manifestation of tubular toxicity present in 12% to 100% of cisplatin-treated patients.^23^ Nevertheless in the present study, using the KDIGO age-dependent phosphate values,^19^ hypophosphatemia was more frequent (65%) and persistent than hypomagnesemia (40.7%) in cisplatin-treated children. Ariceta et al^23^ demonstrated that all children treated with cisplatin present increased magnesiuria immediately after therapy, with a minimal dose to induce hypomagnesemia of 300 mg/m^2^BSA. We found that cisplatin total dose had a significant negative correlation with magnesium serum levels after 12 months of treatment.

We found that patients with nephrotoxicity have impairment in longitudinal growth manifested as statistically significant worsening on the height Z Score at 12 months after treatment, interestingly, patients with nephrotoxicity Grade 1 (asymptomatic, not treated) were the most affected.

Our study has limitations such as the retrospective design, the small number of patients, single center, the lack of a control group of children with solid tumors not treated with platinum compounds, and absence of information about Tanner stage and nutritional status. The strength is that all patients had serum electrolyte and creatinine results before each cisplatin cycle and in the 12-month follow-up period.

Cisplatin was introduced in medical practice in 1970. The importance of hydration and electrolyte supplementation during infusion to prevent adverse events was described and integrated to management protocols in the 1980s and 1990s. During the study period (2002–2013) cisplatin infusion itself was provided in similar way, nevertheless there have been other improvements in the treatment of cancer patients at our Institution such as the related to surgical techniques, the use of filgastrim for neutropenia and the availability of a linear accelerator for targeted radiation therapy since 2010. Any of these changes could improve cancer outcomes and reduce complete reliance on cisplatin to treat solid tumors.

Childhood and adolescence are crucial times to acquire the peak bone mass. Phosphate is a key component of the hydroxiapatite crystals (Ca_10_(PO4)_6_(OH)_2_) in the bone mineral phase.^24^ Magnesium is also important mineral in bone health, and its depletion is related to low parathyroid hormone and bone demineralization.^25–27^

We suggest that the observed impairment in longitudinal growth in patients with nephrotoxicity may be due to phosphate and magnesium urinary losses; patients with asymptomatic, nontreated electrolyte abnormalities (nephrotoxicity Grade 1) are at higher risk of lack of longitudinal growth, cisplatin total dose has a negative relationship with magnesium serum levels at 12 months. There are studies in animal models that suggest that hypomagnesemia enhances cisplatin accumulation in renal tissue by upregulating the organic cation transporter 2 (OCT2) transporter.^28^ Circulating levels of cisplatin have been shown up to 20 years after therapy, thus long-term renal effects are expected.^15,29^

The molecular mechanisms of cisplatin-induced damage in the nonproliferating renal tubular cells are not fully understood. Cisplatin clearance by the kidney depends upon glomerular filtration and tubular secretion. Cisplatin accumulates in the kidney at higher concentrations than in the blood and other organs, contributing to kidney injury. This high drug concentration is attributed to several membrane transporters (Copper transporter 1 -CTR1/SLC31A1-, copper-transporting ATPase 1 -ATP7A-, copper-transporting ATPase 2 –ATP7B-, multidrug and toxin extrusion protein 1 – MATE1/SLC47A1-, OCT2).^30–32^

Further studies are needed to identify if genetic variants can predict the nephrotoxicity risk, as occur with the ototoxicity,^33,34^ and importantly if electrolyte supplementation can prevent the deleterious effect of nephrotoxicity on longitudinal growth and bone health.
